# Association between Vitamin D and *Candida*-Associated Denture Stomatitis

**DOI:** 10.3390/dj8040121

**Published:** 2020-10-21

**Authors:** Miranda Muhvić-Urek, Ema Saltović, Alen Braut, Daniela Kovačević Pavičić

**Affiliations:** 1 Department of Oral Medicine and Periodontology, Faculty of Dental Medicine, University of Rijeka, Kresimirova 40, 51 000 Rijeka, Croatia; 2 Dental Clinic, Clinical Hospital Center Rijeka, Kresimirova 40, 51 000 Rijeka, Croatia; ema.saltovic@medri.uniri.hr (E.S.); alen.braut@fdmri.uniri.hr (A.B.); daniela.kovacevic@fdmri.uniri.hr (D.K.P.); 3 Department of Restorative Dentistry and Endodontics, Faculty of Dental Medicine, University of Rijeka, Kresimirova 40, 51 000 Rijeka, Croatia; 4 Department of Prosthodontics, Faculty of Dental Medicine, University of Rijeka, Kresimirova 40, 51 000 Rijeka, Croatia

**Keywords:** 25-hydroxyvitamin D, candidiasis, oral, stomatitis, denture, vitamin D

## Abstract

*Candida*-associated denture stomatitis (CADS) is a fungal infection affecting 60–65% of denture wearers. Its etiology is complex and multifactorial and often associated with host immunodeficiency. Evidence exists that vitamin D has potential immunomodulatory and anti-inflammatory effects. The aim of this case–control study was to assess the association between vitamin D levels and CADS. The study included 32 complete denture wearers with CADS and 32 sex- and age-matched complete denture wearers without CADS. The patients were clinically examined, and the severity of denture stomatitis was assessed according to Newton’s classification scale. The serum vitamin D level was determined via the use of an electrochemiluminescence assay. The vitamin D level in the CADS group and control group was 54.68 ± 17.07 and 56.82 ± 17.75 nmol/L, respectively. There was no significant difference between the groups (*p* = 0.622). Univariate logistic regression analysis showed that the presence of CADS was not associated with hypovitaminosis D (odds ratio (OR) = 1.44; 95% confidence interval (CI) = 0.37–5.54). It can be concluded that vitamin D is not associated with CADS and does not play a significant role in host susceptibility to CADS. This finding suggests that vitamin D screening is not indicated routinely in patients with *Candida*-associated denture stomatitis.

## 1. Introduction

*Candida*-associated denture stomatitis (CADS) is a fungal infection of the oral mucosa occurring beneath dentures [1]. It is considered a *Candida*-associated lesion, together with angular cheilitis and median rhomboid glossitis [2,3]. Its main feature is that antifungal therapy alone does not cure this condition, and the removal/treatment of predisposing factors must be included [2].

CADS has been found to occur in 60–65% of denture wearers [4]. Although *Candida (C.) albicans* has been identified as the primary pathogen of CADS, other species such as *C. glabrata*, *C. tropicalis,* and *C. parapsilosis* have been found less frequently [5,6].

According to published data, CADS is the most common form of candidiasis [7]. It presents itself as erythema and inflammatory hyperplasia of the mucosa. Newton [8] classified denture stomatitis in 1962 into three types: punctiform hyperemia (Type I), diffuse hyperemia (Type II), and granular hyperemia (Type III). The changes can be manifested in the partial and complete denture wearers and occur more often in the maxilla.

The etiology of CADS is complex and multifactorial. Several local and systemic predisposing factors may convert *Candida* from normal commensal flora (saprophytic stage) to a pathogenic form, leading to disease onset [4,9,10]. Local factors such as dry mouth, local trauma, complete denture wearing, poor denture hygiene, continuous denture wearing, ill-fitting dentures, carbohydrate-rich diets, and acidic salivary pH favor biofilm accumulation in the oral environment and promote the growth of *Candida* species (spp.), and affect the immune response of oral mucosa [3,4,9,11,12]. On the other hand, systemic factors such as diabetes mellitus, immunosuppression (e.g., chemotherapy, corticosteroids, immunosuppressive drugs, and biological therapies), immunodeficiencies (e.g., HIV infection, acute leukemia and agranulocytosis), nutrition and hematinic deficiencies (e.g., iron, folate, and B12) can have an impact on host defense mechanisms [3,4].

In some cases, despite antimycotic drug use and the removal or treatment of predisposing factors, CADS persists or reactivates [13]. More research pertaining to the identification of potential new local and systematic predisposing factors is, therefore, needed.

Apart from having a major role in mineral metabolism (calcium and phosphate) and bone health, vitamin D plays a role in preventing malignancies, infectious diseases, and chronic inflammatory diseases [14,15,16]. It maintains oral health by controlling bone loss, decreasing bone resorption, as well as preventing infectious and inflammatory disease [14]. Vitamin D has immunomodulatory effects and affects both the innate and adaptive immune systems [15]. Its antimicrobial (antibacterial, antiviral, and antifungal) effects are achieved through various immune cells such as lymphocytes B, lymphocytes T, monocytes, macrophages, and neutrophils [15,17,18], as well as the stimulation of antimicrobial protein secretion [17]. The amount of available data related to vitamin D and fungal infection is low when compared to how it is involved in bacterial and viral infections [17]; furthermore, the clinical data pool concerning these matters is small.

The aim of this case–control study was to assess the association between vitamin D levels and *Candida*-associated denture stomatitis.

## 2. Materials and Methods

### 2.1. Subjects

The study comprised 32 Caucasian complete denture wearers with CADS and 32 sex- and age-matched control subjects (complete denture wearers without any signs of CADS). All participants were patients at the Department of Oral Medicine and at the Department of Prosthodontics at the Dental Clinic, Clinical Hospital Center Rijeka. The inclusion criteria were presence of upper complete denture and first dental visit to our departments. The exclusion criteria were vitamin D replacement therapy, systemic or topical anti-fungal therapy, and/or oral mouthwashes received in the preceding month.

The sample size calculation was based on previous study that evaluated the serum vitamin D level in patients with periodontitis [19], where the vitamin D levels in experimental and control groups were 41.9 ± 16.3 and 56.9 ± 14.2 ng/L, respectively. A total of 19 patients in each group were necessary to reach 80% of statistical power and alpha 0.05.

### 2.2. Questionnaire

The participants were interviewed using a questionnaire that included information about age, gender, and prescribed medications.

### 2.3. Clinical Examination

The clinical data were collected while the patient was seated in a dental chair illuminated with professional dental light and using a set of standard dental instruments. The intraoral examinations were performed by the one of the authors (M.M.-U.). A diagnosis of CADS was made on the basis of clinical features stated in World Health Organization guidelines [20], Burket’s Oral Medicine textbook [3], and microbiological analyses. The clinical severity of denture stomatitis was graded according the Newton’s classification scale into Type I, Type II, and Type III [8].

### 2.4. Cultivation and Identification of Candida spp.

Swabs were taken from participants’ palatal mucosa using sterile swab sticks. The material was cultivated on Sabouraud dextrose agar for 72 h at 37 °C. *Candida* spp. was identified based on germ-tube formation, chlamydospore production, and carbohydrate assimilation using the API ID 32C system (bioMerieux, Marcy l’Etoile, France) [21,22,23].

### 2.5. Serum Vitamin D Measurement

Venous blood sample (3.5 mL) was collected in serum separator tubes in the hospital laboratory by a medical professional. After clotting, the serum was centrifuged at 2000× *g* for 10 min. The total vitamin D level was determined using a Roche Elecsys Vitamin D total electrochemiluminescence assay and a Cobas e601 analyzer (Roche Diagnostics GmbH, Manheim, Germany). The measuring range of the test was 7.50–175 nmol/L. Intra-assay and inter-assay coefficients of variation were below 5%. According to vitamin D levels, participants were categorized into four groups: vitamin D sufficient (>75 nmol/L), vitamin D insufficient (>50–≤75 nmol/L) and moderately (>25–≤50 nmol/L) or severely (≤25 nmol/L) vitamin D deficient [24].

### 2.6. Ethical Considerations

The study protocol was approved by the Ethics Committee of the Clinical Hospital Center Rijeka (Ethical approval code 003-05/20-1/41, Project identification code uniri-biomed-18-65, approval date 10 April 2020). Ethical guidelines set forth in the Declaration of Helsinki were followed. All participants gave their informed consent prior to being included in the study.

### 2.7. Statistical Analysis

Statistical analysis of data was performed using Statistica for Windows, version 12.7 (StatSoft, Inc., Tulsa, OK, USA). The Kolmogorov–Smirnov normality test was applied to data. The Student’s *t*- and Kruskal–Wallis ANOVA tests were applied to analyze age and vitamin D level differences between groups. Chi-square and Fisher’s exact tests were used to compare the differences for categorical values. In order to establish the association between the hypovitaminosis D and CADS, the odds ratio (OR) and its 95% confidence interval (CI) were calculated using a logistic regression model. A *p* value of <0.05 was considered statistically significant.

## 3. Results

### 3.1. Demographic Data

The demographic data of participants are shown in Table 1. There were no differences based on gender and age between the groups (*p* > 0.05 each). Women were more represented than men (female:male ratio, 3.6:1).

### 3.2. Candidal Infection and the Severity of Denture Stomatitis

**Figure 1:**
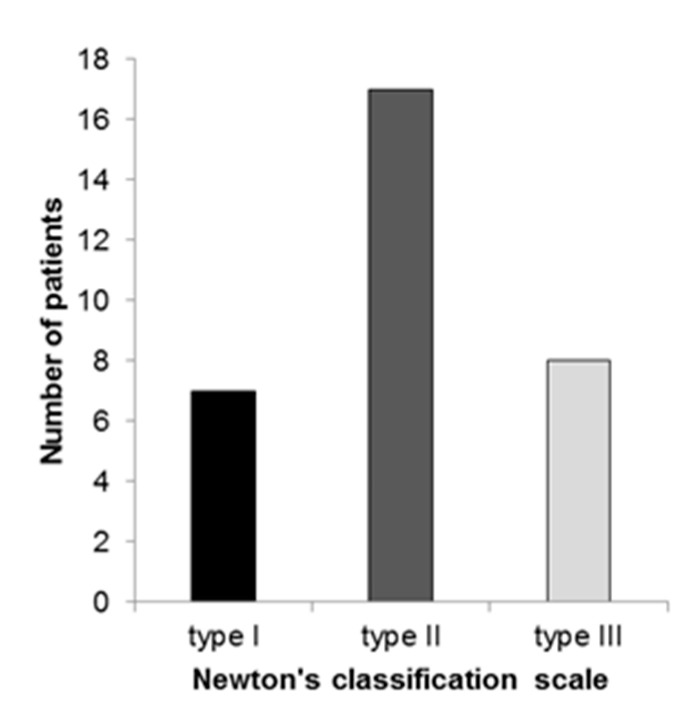
Severity of denture stomatitis in patients with *Candida*-associated denture stomatitis.

*C. albicans* was isolated in 30 (93.73%) patients, while *C. glabrata* was isolated in two (6.26%) patients with CADS. Clinically, according the Newton’s classification scale, Type II was the most prevalent type and presented in 17 (53.12%) patients, followed by Type III and Type I (Figure 1).

### 3.3. Serum Vitamin D Level

The mean value (±standard deviation) of vitamin D level in patients with CADS was 54.68 (±17.07) nmol/L and in control subjects it was 56.82 (±17.75) nmol/L. There was no significant difference between groups (*p* = 0.622). In both groups, female participants had lower vitamin D levels than the male participants, but no statistically significant difference was found (Table 2).

Furthermore, there was no statistically significant difference between the groups in terms of vitamin D level being affected by denture stomatitis severity (Table 3).

### 3.4. Vitamin D Status

Table 4 presents data related to vitamin D status for both of the investigated groups. In the group of patients with CADS, hypovitaminosis D was found in 27 (84.37%) participants. In the control group, hypovitaminosis D was found in 26 (81.25%) participants. Univariate logistic regression analysis showed that the presence of CADS was not associated with hypovitaminosis D (OR = 1.44; 95% CI = 0.37–5.54).

According to gender, in CADS group three women (12%) had normal levels of vitamin D; eight women (32%) had insufficient levels; and 14 (56%) had a deficiency. In the control group, three women (12%) had normal levels of vitamin D, 10 (40%) had insufficient levels, and 12 (48%) had a deficiency. No statistically significant difference was found in terms of vitamin D status between female participants depending on the presence of fungal infection (*p* = 0.83). In the group of men with CADS, two (28.57%) men had normal levels, three (42.86%) had an insufficient level, and two (28.57%) had a vitamin D deficiency. In the control group of men, three (42.86%) men had normal levels, two (28.57%) had insufficient levels, and two (28.57%) had a vitamin D deficiency. No significant difference in vitamin D status was found between these groups (*p* = 0.82).

## 4. Discussion

Vitamin D deficiency is associated with numerous adverse health outcomes [25,26]. Patients with vitamin D deficiency have an increased risk of developing skeletal diseases (e.g., rickets, osteopenia, and osteoporosis) [26,27], cardiovascular diseases (e.g., hypertension, myocardial infarction, and stroke) [25,28,29], autoimmune diseases (e.g., inflammatory bowel disease, multiple sclerosis, rheumatoid arthritis, and diabetes mellitus Type I) [25,30,31,32], cancers (e.g., leukemia, squamous cell carcinoma, breast cancer, and bowel carcinoma) [25,33,34] and infectious diseases (e.g., tuberculosis, viral respiratory infections, and sepsis) [17,25,35,36]. Nowadays, many ongoing studies have set their aim on discovering the association between vitamin D and Coronavirus disease 2019 (COVID-19), i.e., its possible role in the prevention and risk of development this disease [37,38]. However, no clear correlation was found yet on the association between hypovitaminosis D and COVID-19.

In the oral cavity, vitamin D plays an important role in the maintenance of oral health by maintaining bone mass, preventing gingivitis, periodontitis, dental caries and tooth loss, and preventing the onset of malignant and infectious diseases by stimulating immunity and through its antimicrobial properties [39,40,41,42]. Antonoglou et al. [19] presented a low serum level of 1.25(OH)2D in patients with chronic periodontitis. The same group of researchers demonstrated that periodontal therapy increases the serum levels of vitamin D in patients with diabetes mellitus Type 1 [43]. It is considered that vitamin D reduces the likelihood of gingivitis, due to its anti-inflammatory action [39]. Vitamin D insufficiency through its negative influences on the density of alveolar bone and the immune system, contributes to infection and inflammation, which lead to periodontitis. Studies have shown that vitamin D has an antifungal effect by stimulating keratinocytes and macrophages [44] and via the production of antimicrobial proteins such as cathelicidin [45,46] and beta-defensins [47].

Candidiasis is the most common oral fungal infection, particularly in immunocompromised patients, the elderly, and denture wearers. Sroussi et al. [48] determined that vitamin D deficiency is an important predictor for the onset of oral candidiasis in patients with HIV infection. They also determined that vitamin D inversely affects calprotectin, an antimicrobial and immune regulatory protein complex that influences neutrophil function and inhibit its oxidative functions. A recently published study by Lim et al. [18] suggests that candidemic patients have lower vitamin D concentrations than hospitalized patients and healthy subjects.

The hypothesis for this study was that the hypovitaminosis D in patients with dentures is one of predisposing factors for onset of CADS. To the best of our knowledge, this is the first study that investigated serum vitamin D level in patients with CADS. We determined that patients with CADS do not have significantly lower vitamin D serum levels compared to control subjects. However, the female subjects of both groups had lower vitamin D serum levels than their male counterparts, a finding supported by other authors [49,50,51]. We also found a trend toward lower serum vitamin D level with increasing CADS severity. This finding is suggestive of an immunomodulatory role of vitamin D in severe types of CADS. In order to confirm this finding, a larger sample size is needed.

There are contradictory data for the influence of vitamin D in the treatment of fungal infections. Bonilla [52] described, in 1954, three clinical cases of severe refractory chromoblastomycosis, where treatment with 600,000 IU of calciferol led to the significant improvement of cutaneous lesions. Cantorna et al. [53] published data showing that vitamin D had no effect on systemic candidiasis. In an experimental study using a mouse model, Lim et al. [18] indicated the therapeutic effect of vitamin D in the treatment of systematic candidiasis, depending on dose. A low dosage had the best results, while high doses were not effective and even suppressed the mice’s immune responses.

The limitation of this study is small number of participants especially male participants. Studies with a larger sample size may clarify the association of lower vitamin D level and severe types of CADS and bring up more accurate findings. Future studies are still warranted for discovering other predisposing factors for CADS occurrence, as well as the role of vitamin D in other types of *Candida* infections.

## 5. Conclusions

It can be concluded that vitamin D has no significant role in terms of being a systemic, predisposing factor in fungal infection that occurs among denture wearers. This indicates that vitamin D determination during the diagnostics and treatment of *Candida*-associated denture stomatitis is not necessary.

## Figures and Tables

**Table 1:** Demographic data of subjects in *Candida*-associated denture stomatitis and control groups.

Demographic Data	Groups		*p* Value
	CADS	Control	
Gender			
Female/*n* (%)	25 (78.13)	25 (78.13)	*p* = 1 *
Male/*n* (%)	7 (21.87)	7 (21.87)	
Age/years			
Average	68.9	68.9	*p* = 1 **
Standard deviation	8.4	8.4	
Min.	53	53	
Max.	83	83	

CADS, *Candida*-associated denture stomatitis. * Chi-square test. ** Student’s *t*-test.

**Table 2:** Serum vitamin D level according the groups and gender.

	Groups				
	CADS		Control		*p* Value
	(*n* = 32)		(*n* = 32)		
Vitamin D/nmol/L					
mean ± SD	54.68 ± 17.07		56.82 ± 17.75		*p* = 0.622 *
95% CI	48.53–60.83		50.49–63.14		
median	female	male	female	male	
(5th–95th percentile)	(*n* = 25)	(*n* = 7)	(*n* = 25)	(*n* = 7)	
	48.2	69.7	51.2	66.57	*p* = 0.16 **
	(30.4–78.8)	(42.1–83.7)	(30.4–78.5)	(41–98.2)	

CADS, *Candida*-associated denture stomatitis; CI, confidence interval. * Student’s *t*-test. ** Kruskal–Wallis ANOVA test.

**Table 3:** Serum vitamin D level depending on denture stomatitis severity.

	Groups				*p* Value
	Type I DS	Type II DS	Type III DS	Control	
	(*n* = 7)	(*n* = 17)	(*n* = 8)	(*n* = 32)	
Vitamin D/nmol/L					
median	57.4	50.3	43.4	54.35	*p* = 0.601 *
(5th–95th percentile)	(33.50–78.80)	(36.50–83.70)	(26.80–74.80)	(30.40–80.60)	

DS, denture stomatitis. * Kruskal–Wallis ANOVA test.

**Table 4:** Vitamin D status in *Candida*-associated denture stomatitis and control groups.

Vitamin D Status	Groups		
	CADS	Control	*p* Value
	*n* (%)	*n* (%)	
sufficiency (>75 nmol/L)	5 (15.63)	6 (18.75)	
insufficiency (>50–≤75 nmol/L)	11 (34.77)	13 (40.625)	*p* = 0.75 *
moderate deficiency (>25–≤50 nmol/L)	16 (50)	13 (40.625)	
severe deficiency (≤25 nmol/L)	0	0	

CADS, *Candida*-associated denture stomatitis. * Chi-square test.
